# Development of phospho-specific Rab protein antibodies to monitor *in vivo* activity of the LRRK2 Parkinson's disease kinase

**DOI:** 10.1042/BCJ20170802

**Published:** 2018-01-02

**Authors:** Pawel Lis, Sophie Burel, Martin Steger, Matthias Mann, Fiona Brown, Federico Diez, Francesca Tonelli, Janice L. Holton, Philip Winglok Ho, Shu-Leong Ho, Meng-Yun Chou, Nicole K. Polinski, Terina N. Martinez, Paul Davies, Dario R. Alessi

**Affiliations:** 1 MRC Protein Phosphorylation and Ubiquitylation Unit, School of Life Sciences, University of Dundee, Dundee DD1 5EH, U.K.; 2 Department of Proteomics and Signal Transduction, Max-Planck-Institute of Biochemistry, Martinsried, Germany; 3 Division of Neuropathology, UCL Institute of Neurology, Queen Square, London, U.K.; 4 Division of Neurology, Department of Medicine, University of Hong Kong, Pok Fu Lam, Hong Kong; 5 Abcam, 863 Mitten Rd, Burlingame, CA 94010, U.S.A.; 6 The Michael J. Fox Foundation for Parkinson's Research, Grand Central Station, PO Box 4777, New York, NY 10163, U.S.A.

**Keywords:** antibodies, leucine-rich repeat kinase, Parkinson's disease, protein kinase, Rab GTPases, Rab10

## Abstract

Mutations that activate the LRRK2 (leucine-rich repeat protein kinase 2) protein kinase predispose to Parkinson's disease, suggesting that LRRK2 inhibitors might have therapeutic benefit. Recent work has revealed that LRRK2 phosphorylates a subgroup of 14 Rab proteins, including Rab10, at a specific residue located at the centre of its effector-binding switch-II motif. In the present study, we analyse the selectivity and sensitivity of polyclonal and monoclonal phospho-specific antibodies raised against nine different LRRK2-phosphorylated Rab proteins (Rab3A/3B/3C/3D, Rab5A/5B/5C, Rab8A/8B, Rab10, Rab12, Rab29[T71], Rab29[S72], Rab35 and Rab43). We identify rabbit monoclonal phospho-specific antibodies (MJFF-pRAB10) that are exquisitely selective for LRRK2-phosphorylated Rab10, detecting endogenous phosphorylated Rab10 in all analysed cell lines and tissues, including human brain cingulate cortex. We demonstrate that the MJFF-pRAB10 antibodies can be deployed to assess enhanced Rab10 phosphorylation resulting from pathogenic (R1441C/G or G2019S) LRRK2 knock-in mutations as well as the impact of LRRK2 inhibitor treatment. We also identify rabbit monoclonal antibodies displaying broad specificity (MJFF-pRAB8) that can be utilised to assess LRRK2-controlled phosphorylation of a range of endogenous Rab proteins, including Rab8A, Rab10 and Rab35. The antibodies described in the present study will help with the assessment of LRRK2 activity and examination of which Rab proteins are phosphorylated *in vivo*. These antibodies could also be used to assess the impact of LRRK2 inhibitors in future clinical trials.

## Introduction

Commonly occurring autosomal-dominant missense mutations within the LRRK2 (leucine-rich repeat protein kinase 2) protein are linked to Parkinson's disease [1,2]. Mutations in LRRK2 are observed in ∼1% of sporadic and ∼5% of familial Parkinson's patients [3]. LRRK2 is a large protein containing numerous domains including a ROC/COR-type GTPase and a serine/threonine protein kinase [4]. The most frequent pathogenic mutation (G2019S) is located within the kinase domain and directly enhances kinase activity, suggesting that LRRK2 inhibitors might offer therapeutic benefit for preventing and treating Parkinson's disease [5–8]. Because of this, several pharmaceutical companies have generated highly selective, potent and brain-penetrable LRRK2 inhibitors that are in the late stage of preclinical development [9].

Recent studies have identified key physiological substrates of LRRK2 as members of the Rab GTPase family including Rab8A and Rab10 [10–13]. LRRK2 phosphorylates Rab proteins at an evolutionarily conserved Ser/Thr site lying within the effector-binding, switch-II motif (e.g. Thr72 for Rab8A and Thr73 for Rab10) [10]. All clear-cut LRRK2 pathogenic mutations tested, including the G2019S mutant, as well as the R1441G/C and Y1699C mutations located within the ROC/COR GTPase domain, markedly elevate Rab10 phosphorylation in cell lines (HEK293 and mouse embryonic fibroblasts) as well as mouse tissues (the brain, spleen, lung and kidney) [10,11].

Recent work has revealed that 14 Rab proteins (Rab3A/B/C/D, Rab5A/B/C, Rab8A/B, Rab10, Rab12, Rab29, Rab35 and Rab43) are phosphorylated by LRRK2 in a HEK293 cell overexpression system [12]. Moreover, at least 23 other Rab proteins that are not LRRK2 substrates are also phosphorylated at the equivalent switch-II phosphorylation site [12]. These include Rab1, which is phosphorylated at the switch-II motif site by TAK1 kinase (Thr75) [14], as well as Rab7 (Ser72) that is phosphorylated by an unknown kinase during endosome maturation [15,16]. Thus, at least 37 of the 70 Rab proteins are phosphorylated within the effector-binding switch-II motif, and this is likely to represent a major mechanism by which Rab proteins are controlled.

**Figure 1.**
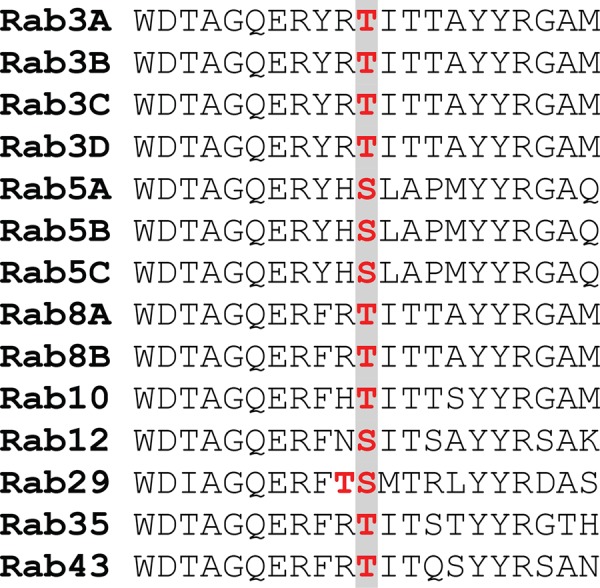
Sequence alignment encompassing the phosphorylation sites of the 14 Rab proteins phosphorylated by LRRK2. Phosphorylation sites marked in red. Sequence shown is human, but is identical in most other mammals including mouse.

Endogenous Rab protein phosphorylation has until now been largely assessed by mass spectrometry [10,12,13] or by the ability of the Phos-tag reagent to retard the electrophoretic mobility of LRRK2-phosphorylated Rab proteins [11]. Polyclonal antibodies to various LRRK2 Rab protein phosphorylation sites have also been raised [10,13], but the selectivity of these antibodies towards the various Rab proteins phosphorylated by LRRK2 has not been rigorously assessed. As the residues encompassing the switch-II phosphorylation sites on the Rab proteins are highly similar (Figure 1), it is likely that polyclonal antibodies raised against these sites will cross-react with multiple phosphorylated Rab proteins, thus complicating analysis.

In this study, we describe our efforts to develop polyclonal and monoclonal antibodies towards LRRK2-phosphorylated Rab proteins and our approach to rigorously assess antibody specificity and potency. This effort resulted in the development of a series of sensitive and highly selective rabbit monoclonal phospho-antibodies (MJFF-pRAB10) that detect endogenous LRRK2-phosphorylated Rab10, in all cell lines and tissues we have analysed, including human brain. We also identify rabbit monoclonal antibodies (MJFF-pRAB8) that efficiently immunoprecipitate a subgroup of endogenous LRRK2-phosphorylated Rab proteins (Rab8A, Rab10 and Rab35) that can therefore be deployed to assess LRRK2-mediated phosphorylation of multiple endogenous Rab proteins. The antibodies described in the present study will be valuable tools to assess the impact that inhibitors and pathogenic mutations have on LRRK2 activity *in vivo*.

## Materials and methods

### Material

MLi-2 inhibitor was synthesised by Natalia Shpiro (University of Dundee) as described recently [17,18]. All peptide antigens were synthesised by ‘peptides and elephants’ (http://www.peptides.de/) and HPLC-purified and stored in aliquots in sealed glass ampules in argon to avoid oxidation. For each phospho-peptide antigen, we also ordered a dephosphorylated version that was used for negative affinity purification or for inclusion in immunoblotting conditions (sheep polyclonal antibodies only).

### Sheep polyclonal antibodies

Table 1 summarises the sheep phospho-specific and total Rab polyclonal antibodies generated for the present study. It includes details of the antigens utilised and sheep numbers for each antibody programme. An additional N-terminal Cys residue was added to each peptide antigen to conjugate the peptide to both keyhole limpet haemocyanin (KLH) and bovine serum albumin [19]. Sheep were immunised with each antigen followed by up to 12 further injections 28 days apart, with bleeds performed 7 days after each injection. Antibodies were affinity-purified from serum using the same phospho-peptides or Rab protein antigen that they were raised against [19]. For total antibodies, a recombinant Rab protein with a different epitope tag than that used for immunisation was employed for affinity purifications (Table 1). All sheep affinity-purified antibodies were used for immunoblotting at final concentrations of 1 µg/ml (in 5% non-fat milk in TBS-T), and for phospho-antibodies, immunoblotting was conducted in the presence of 10 µg/ml of non-phosphorylated peptide antigen in order to sequester any non-phospho-specific antibody. All bleeds of each antibody were evaluated and data are summarised in Supplementary Figure S1. All sheep polyclonal antibodies generated for the present study can be requested via our reagents website (https://mrcppureagents.dundee.ac.uk/).

**Table 1:** Antigens employed for sheep polyclonal phospho-specific and total Rab antibodies Phosphorylated serine/threonine marked with S*/T*.

Antibody	Sheep number	Antigen
Rab3 pThr86	SA082	CAGQERYRT*ITTAYYR
Rab5 pSer84	S942D	CAGQERYHS*LAPMYYR
Rab7L1 (Rab29) pThr71	S877D	CIAGQERFT*SMTRLYYR
Rab7L1 (Rab29) pSer72	SA136	CDIAGQERFTS*MTRLYYRSS
Rab8 pThr72	S874D	CAGQERFRT*ITTAYYR
Rab10 pThr73	S873D	CAGQERFHT*ITTSYYR
Rab12 pSer106	S876D	CAGQERFNS*ITSAYYR
Rab35 pThr72	SA083	CAGQERFRT*ITSTYYR
Rab43 pThr82	SA334	CAGQERFRT*ITQSYYR
Rab12 total	SA227	Rab12 (no epitope tag) (affinity-purified with 6HIS-SUMO-Rab12)
Rab35 total	SA314	GST-Rab35 (affinity-purified with MBP-Rab35)
Rab43 total	SA135	GST-Rab43 (affinity-purified with HIS-SUMO-Rab43)

### Rabbit polyclonal and monoclonal antibodies

Rabbit immunisation and rabbit antibody generation were performed by Abcam, Inc. (Burlingame, CA). Eight New Zealand White rabbits were immunised with target peptides described in Table 2 for each antibody using a standard protocol of five injections and two bleedings for each rabbit. Three subcutaneous injections were performed using the KLH immunogens, followed by two subcutaneous injections using the ovalbumin immunogens. At the time of each injection, an immunogen aliquot was thawed and combined with Complete Freund's Adjuvant (initial immunisation) or with incomplete Freund's Adjuvant (for the subsequent injections). Serum bleeds of 25 ml were obtained after the fourth and fifth immunisations (50 ml total). Antibodies were affinity-purified at this stage and evaluated in parallel with serum. One rabbit producing the best antibody was chosen for monoclonal antibody generation and was intravenously boosted with immunogen 4 days before splenectomy. Hybridoma fusion was performed according to the established protocol with minor modifications [20]. Briefly, splenocytes were harvested from the immunised rabbit and fused with rabbit plasmacytoma cells 240E-W2 [21] using PEG4000 (Sigma Chemical, St. Louis, MO) and selected by HAT (hypoxanthine, aminopterin and thymidine). At the end of the selection, hybridoma clones growing in the original 96-well plates were transferred to 24-well plates with a medium change. Hybridoma supernatants were collected and screened for antigen binding by enzyme-linked immunosorbent assay (ELISA) and western blot. Hybridomas that were specific in both assays were subcloned, expanded and frozen for future use. All rabbit polyclonal and monoclonal antibodies were diluted in 5% BSA (bovine serum albumin) in TBS-T. The MJFF-pRab8 and MJFF-pRab10 rabbit monoclonal antibodies will be available commercially from Abcam (www.abcam.com) in 2018. Table 3 lists the MJFF antibodies analysed in the present study with future Abcam catalogue number.

**Table 2:** Antigens employed for rabbit polyclonal and monoclonal phospho-specific Rab antibodies Phosphorylated serine/threonine marked with S*/T*.

Antibody	Abcam project number	Antigen	Rabbit numbers	Name of final monoclonal antibodies
Rab8 pThr72	MJF-20	C-Ahx-AGQERFRT*ITTAYYR-amide	E8262-E8269	MJFF-pRab8
		Ac-AGQERFRT*ITTAYYR-Ahx-C		
Rab10 pThr73	MJF-21	C-Ahx-AGQERFHT*ITTSYYR-amide	E8254-E8261	MJFF-pRab10
		Ac-AGQERFHT*ITTSYYR-Ahx-C

**Table 3:** MJFF-pRab8 and MJFF-pRab10 monoclonal phospho-specific Rab antibodies

MJFF clone name	Abcam catalogue number
MJFF-pRab8^clone-1^	MJF-20-25-2
MJFF-pRab8^clone-2^	MJF-20-55-4
MJFF-pRab8^clone-3^	MJF-20-19-6
MJFF-pRab8^clone-4^	MJF-20-19-3
MJFF-pRab8^clone-5^	MJF-20-25-5
MJFF-pRab8^clone-6^	MJF-20-38-1
MJFF-pRab8^clone-7^	MJF-20-55-3
MJFF-pRab8^clone-8^	MJF-20-58-2
MJFF-pRab8^clone-9^	MJF-20-58-6
MJFF-pRab10^clone-1^	MJF-21-108-10
MJFF-pRab10^clone-2^	MJF-21-83-4
MJFF-pRab10^clone-3^	MJF-21-136-1
MJFF-pRab10^clone-4^	MJF-21-108-1
MJFF-pRab10^clone-5^	MJF-21-22-5
MJFF-pRab10^clone-6^	MJF-21-233-3
MJFF-pRab10^clone-7^	MJF-21-250-7
MJFF-pRab10^clone-8^	MJF-21-279-2
MJFF-pRab10^clone-9^	MJF-21-279-6

### Commercial antibodies

Anti-α-tubulin (#5174), anti-HA (#3724), anti-Rab8A (#6975), anti-Rab10 (#8127) and anti-phospho-p44/42 MAPK (Erk1/2) (#9101) were from Cell Signaling Technologies. Anti-ERK1 (C16) was from Santa Cruz (sc-93). Mouse anti-LRRK2 antibody was from Antibodies, Inc. (#75-253). Rabbit monoclonal antibodies for total LRRK2 (UDD3) and pS935-LRRK2 (UDD2) were purified at the University of Dundee as described previously [22]. All commercially available antibodies were diluted in 5% BSA in TBS-T. The MJFF-total Rab10 antibody [23] generated by nanoTools (www.nanotools.de) will be commercialised by the Michael J Fox Foundation in early 2018.

### Plasmids

Constructs for overexpression of LRRK2 and Rab employed in the present study were generated at the University of Dundee. Constructs utilised were HA-Rab3A (DU51539), HA-Rab3B (DU55007), HA-Rab3C (DU55048), HA-Rab3D (DU26388), HA-Rab5A (DU26389), HA-Rab5B (DU26106), HA-Rab5C (DU47956), HA-Rab8A (DU35414), HA-Rab8B (DU39856), HA-Rab10 (DU44250), HA-Rab12 (DU48963), HA-Rab29 (DU50222), HA-Rab35 (DU26478), HA-Rab43 (DU26392), flag-LRRK2-WT (DU6841) and flag-LRRK2-Y1699C (DU13165). Data sheets for each construct can be viewed and constructs requested on our reagents website (https://mrcppureagents.dundee.ac.uk/).

### Mice

All animal studies were ethically reviewed and carried out in accordance with Animals (Scientific Procedures) Act 1986 and regulations set by the University of Dundee and the U.K. Home Office were strictly adhered to. Animal studies and breeding were approved by the University of Dundee ethical committee and performed under a U.K. Home Office project licence. Mice were multiply housed at an ambient temperature (20–24°C) and humidity (45–55%) maintained on a 12 h light/12 h dark cycle, with free access to food (SDS RM No. 3 autoclavable) and water. The LRRK2[R1441G] knock-in mice were described previously [24]. The LRRK2[R1441C] knock-in mice were obtained from Jackson Laboratory (JAX stock #009347 [25]) and maintained on a C57BL/6J background. Mice were maintained under specific pathogen-free conditions at the University of Dundee (U.K.). Genotyping of mice was performed by PCR using genomic DNA isolated from ear biopsies. Primer 1 (5′-CTGCAGGCTACTAGATGGTCAAGGT-3′) and Primer 2 (5′-CTAGATAGGACCGAGTGTCGCAGAG-3′) were used to detect the wild-type and knock-in alleles. The PCR programme consisted of 5 min at 95°C, then 35 cycles of 30 s at 95°C, 30 s at 60°C and 30 s at 72°C, and 5 min at 72°C. DNA sequencing was used to confirm the knock-in mutation and performed by DNA Sequencing & Services (MRC-PPU; http://www.dnaseq.co.uk) using Applied Biosystems Big-Dye version 3.1 chemistry on an Applied Biosystems model 3730 automated capillary DNA sequencer.

For experiments shown in Figures 5 and 6, age-matched wild-type and LRRK2 knock-in mice (3–6 months of age) were injected subcutaneously either with vehicle [40% (w/v) (2-hydroxypropyl)-β-cyclodextrin (Sigma–Aldrich)] or MLi-2 dissolved in vehicle at 3 mg/kg dose and killed by cervical dislocation 60 min after treatment. Tissues were rapidly isolated and snap-frozen in liquid nitrogen. MLi-2 (10 mg/kg) was used for R1441C mice.

### Cell culture, transfection, treatments and lysis

HEK293 and A549 cells were cultured in Dulbecco's modified Eagle medium (Glutamax and Gibco) supplemented with 10% foetal calf serum, 100 U/ml penicillin and 100 µg/ml streptomycin. For culturing of mouse embryonic fibroblasts (MEFs), the medium was additionally supplemented with non-essential amino acids (Life Technologies) and 1 mM sodium pyruvate (Life Technologies). All cells were regularly tested for mycoplasma contamination. Transient transfections of HEK293 cells were performed 24–48 h prior to cell lysis using polyethylenimine PEIMax (Polysciences). For PEI transfections on a 10 cm diameter dish of ∼70% confluent HEK293 cells, 1 ml of Dulbecco's modified Eagle medium was mixed with 6.6 µg of plasmid DNA and 20 µl of polyethylenimine (0.1% w/v), and incubated at room temperature for 30 min. For co-transfections of LRRK2 and Rab proteins, we typically transfected using 5.3 µg of LRRK2 construct and 1.3 µg of Rab construct (i.e. ratio of 4:1). This DNA–polyethylenimine mixture was added dropwise to the cells. Prior to cell lysis, cells were washed with TBS (20 mM Tris–HCl, pH 7.5, and 150 mM NaCl) on ice and lysed in an ice-cold lysis buffer containing 50 mM Tris–HCl, pH 7.5, 1% (v/v) Triton X-100, 1 mM EGTA, 1 mM sodium orthovanadate, 50 mM NaF, 0.1% (v/v) 2-mercaptoethanol, 10 mM 2-glycerophosphate, 5 mM sodium pyrophosphate, 0.1 µg/ml microcystin-LR (Enzo Life Sciences), 270 mM sucrose and complete EDTA-free protease inhibitor cocktail (Sigma–Aldrich Cat # 11836170001). Lysates were centrifuged at 20 800 ***g*** for 15 min at 4°C and supernatants were used for the Bradford assay (Thermo Scientific) and immunoblot analysis.

### Generation of mouse embryonic fibroblasts

Littermate-matched wild-type and homozygous mutant mouse embryonic fibroblasts (MEFs) were isolated from mouse embryos at day E12.5 as described previously [26]. The LRRK2[R1441C] knock-in mice were obtained from The Jackson Laboratory. The LRRK2[R1441G] knock-in MEFs were described recently [11]. LRRK2[G2019S] knock-in MEFs were generated from knock-in mice originally provided by Eli Lilly that are now distributed through Taconic (line 13940). LRRK2[G2019S] MEFs were generated from the Eli Lilly mice as described recently [10]. Littermate-matched wild-type and homozygous knock-out MEFs were isolated from LRRK2 knock-out mice [22] as described previously [27]. All knock-in and knock-out cell lines were verified by allelic sequencing.

### Generation of CRISPR–Cas9 knock-out cell lines

The A549 Rab8A knock-out and Rab10 knock-out cell lines have been described previously [11, 12].

### Mouse tissue lysate preparation

Frozen mouse tissues were weighed and added to a 10-fold volume excess of ice-cold lysis buffer containing 50 mM Tris–HCl, pH 7.5, 1% (v/v) Triton X-100, 1 mM EGTA, 1 mM sodium orthovanadate, 50 mM NaF, 0.1% (v/v) 2-mercaptoethanol, 10 mM 2-glycerophosphate, 5 mM sodium pyrophosphate, 0.1 µg/ml microcystin-LR (Enzo Life Sciences), 270 mM sucrose and complete EDTA-free protease inhibitor cocktail (Sigma–Aldrich Cat # 11836170001), and homogenised using a POLYTRON homogenizer (KINEMATICA) on ice (5 s homogenisation, 10 s interval and 5 s homogenisation). Lysates were centrifuged at 20 800 ***g*** for 30 min at 4°C and supernatants were used for Bradford assay and immunoblot analysis. Protein lysate (10–40 mg) was obtained from each tissue sample.

### Human brain lysate preparation

Human brain samples were provided by the Queen Square Brain Bank for Neurological Disorders (UCL, London). Frozen human cingulate cortex samples were weighed and added to a 10-fold volume excess of ice-cold lysis buffer containing 50 mM Tris–HCl pH 7.5, 1% (v/v) Triton X-100, 1 mM EGTA, 1 mM sodium orthovanadate, 50 mM sodium fluoride, 10 mM β-glycerophosphate, 5 mM sodium pyrophosphate, 0.1 µg/ml Microcystin-LR (Enzo Life Sciences), 270 mM sucrose and complete EDTA-free protease inhibitor cocktail ((Sigma–Aldrich Cat # 11836170001), and homogenised using POLYTRON homogenizer (KINEMATICA) on ice (5 s homogenisation, 10 s interval and 5 s homogenisation). Lysates were centrifuged at 20 800 ***g*** for 10 min at 4°C. Supernatants were collected, quantified by the Bradford assay (Thermo Scientific) and subjected to immunoblot analysis.

### Immunoblot analysis

Cell or tissue lysates were prepared in SDS–PAGE sample buffer [50 mM Tris–HCl, pH 6.8, 2% (w/v) SDS, 10% (v/v) glycerol, 0.02% (w/v) Bromophenol Blue and 1% (v/v) 2-mercaptoethanol] and heated at 95°C for 5 min. Electrophoresis was undertaken using the NuPAGE Bis–Tris 4–12% gradient gels (Life Technologies) run at 150 V. Proteins were then transferred onto the nitrocellulose membrane (GE Healthcare, Amersham Protran Supported 0.45 µm NC) at 90 V for 90 min. Membranes after transfer were blocked with 5% (w/v) non-fat dry milk dissolved in TBS-T [20 mM Tris–HCl, pH 7.5, 150 mM NaCl and 0.1% (v/v) Tween 20] at room temperature for 45 min. Membranes were then incubated with primary antibodies diluted in 5% BSA (bovine serum albumin) in TBS-T (rabbit polyclonal and monoclonal antibodies) or in 5% non-fat milk in TBS-T (sheep polyclonal antibodies) overnight at 4°C. After washing membranes in TBS-T, membranes were incubated at room temperature for 1 h with either HRP-labelled secondary antibody (Thermo Fisher Scientific #31480, #31460, #31430) diluted (1 : 2500) in 5% non-fat dry milk/TBS-T or with near-infrared fluorescent IRDye antibodies (LI-COR #925-68070, #925-32211) diluted (1 : 20 000) in TBS-T (without milk or BSA). The membranes, after washing in TBS-T, were developed either using ECL [Amersham ECL Western Blotting Detection Reagents (GE Healthcare)] or using the LI-COR Odyssey CLx Western Blot imaging system.

### Phos-tag SDS–PAGE

Phos-tag SDS–PAGE was performed following the general protocol recently described [11], with modified concentration of the Phos-tag reagent used (100 µM Phos-tag acrylamide and 200 µM MnCl_2_). The membranes were developed using ECL [Amersham ECL Western Blotting Detection Reagents (GE Healthcare)].

### Immunoprecipitation of Rab proteins

For the endogenous Rab immunoprecipitations from LRRK2[R1441C/G] and wild-type MEF ± MLi2, the cells were treated with or without 100 nM MLi-2 for 90 min and lysed in an ice-cold lysis buffer containing 50 mM Tris–HCl, pH 7.5, 1% (v/v) Triton X-100, 10% (v/v) glycerol, 1 mM sodium orthovanadate, 50 mM NaF, 10 mM 2-glycerophosphate, 5 mM sodium pyrophosphate, 0.1 µg/ml microcystin-LR (Enzo Life Sciences) and complete EDTA-free protease inhibitor cocktail (Sigma–Aldrich Cat # 11836170001).

The rabbit polyclonal and monoclonal Rab8 phospho-Thr72 antibodies were covalently linked to Protein A-agarose beads using dimethyl pimelimidate [19], at the ratio of 0.2 µg of antibody per 1 µl of beads for monoclonal antibody or 1 µg of antibody per 1 µl of beads for polyclonal antibody. Immunoprecipitations were conducted by incubating 50 µl of conjugated antibody resin, washed in lysis buffer, with 5 mg of MEF extract. After 2 h at 4°C, the beads were then washed three times with phosphate-buffered saline and incubated with 20 µl of 2× SDS–PAGE sample buffer for 2 min at room temperature. The suspension was then centrifuged through a 0.22 µm Spinex filter and the eluate heated to 95°C for 10 min and subjected to immunoblot analysis with diverse Rab protein total antibodies.

For the immunoprecipitation of endogenous Rab12 from MEF lysates prepared as described above, sheep polyclonal Rab12 (SA227) antibody was cross-linked to Protein-G-agarose beads at the ratio of 1 µg of antibody per 2 µl of beads using dimethyl pimelimidate [19]. Immunoprecipitations were conducted by incubating 50 µl of conjugated antibody resin washed in lysis buffer with 5 mg of MEF extract. After 2 h incubation at 4°C, the beads were washed three times with phosphate-buffered saline and incubated with 50 µl of 2× SDS–PAGE sample buffer for 2 min at room temperature. The suspension was centrifuged through a 0.22 µm Spinex filter, and the eluate was heated to 95°C for 10 min and subjected to immunoblot analysis with the Rab phospho-Rab12 pS106 (S876D) antibody.

### *In vitro* phosphorylation of Rab8A and Rab10 by LRRK2

Recombinant Rab8A and Rab10 were expressed and purified as described previously [10, 11]. Purified proteins were phosphorylated using full-length LRRK2[G2019S] (Invitrogen) in a buffer containing 50 mM Tris–HCl, pH 7.5, 0.1 mM EGTA, 10 mM MgCl_2_ and 1 mM ATP (50 µl total volume). A reaction where no LRRK2 was added was also included as a negative control. The kinase reactions were carried out for 4 h at room temperature in Dispo-Biodialysers of 1 kDa molecular mass cut-off (Sigma–Aldrich) put in 0.5 l of the same buffer to allow for the removal of kinase reaction product ADP and to maintain a high level of ATP, in order to maximise phosphorylation stoichiometry of Rab proteins. Kinase reactions were terminated by the addition of SDS–PAGE sample containing 1% (by vol) 2-mercaptoethanol. Known amounts of Rab samples were subjected to Phos-tag SDS–PAGE to assess the stoichiometry of Rab8A/Rab10 phosphorylation. This was used to calculate the amount of phosphorylated Rab proteins and for the potency of MJFF-pRab8 (Figure 8C) and MJFF-pRab10 (Figure 4C) to be assessed.

## Results

### Amino acid sequences encompassing Rab protein LRRK2 phosphorylation sites

The sequences encompassing the phosphorylation site of the 14 Rab proteins that are phosphorylated by LRRK2 are shown in Figure 1. Rab29 (also known as Rab7L1) is unique, as in addition to Ser72 that lies at the equivalent position to other LRRK2 Rab protein phosphorylation sites, possesses an adjacent Thr71 residue that is also phosphorylated by LRRK2 [12]. The sequences within Rab3 isoforms (Rab3A/Rab3B/Rab3C and Rab3D), Rab5 isoforms (Rab5A/Rab5B and Rab5C) and Rab8 isoforms (Rab8A and Rab8B) are identical (Figure 1). Therefore, there are 10 sets of unique sequences, differing by at least one residue that LRRK2 phospho-specific antibodies can be raised against, namely Rab3, Rab5, Rab8, Rab10, Rab12, Rab29 (Thr71), Rab29 (Ser72), Rab29 (Thr71 + Ser72), Rab35 and Rab43 (Figure 1). It should be noted that only for Rab43, the sequence motif that surrounds the LRRK2 phosphorylation site is identical with the sequence surrounding Thr81 in Rab19 that is not thought to be an LRRK2 substrate [12]. Six of the Rab protein LRRK2 phosphorylation sites are on threonine residues (Rab3, Rab8, Rab10, Rab29[Thr71], Rab35 and Rab43) and three on serine residues (Rab5, Rab12 and Rab29 [Ser72]), which could influence the selectivity of the phospho-specific antibodies (Figure 1). The sequences surrounding each of the Rab protein LRRK2 phosphorylation sites are identical in most mammals, including human and mouse, indicating that phospho-specific antibodies will have utility in analysing LRRK2 signalling in a broad range of species.

### Polyclonal phospho-specific Rab protein antibodies

**Figure 2.**
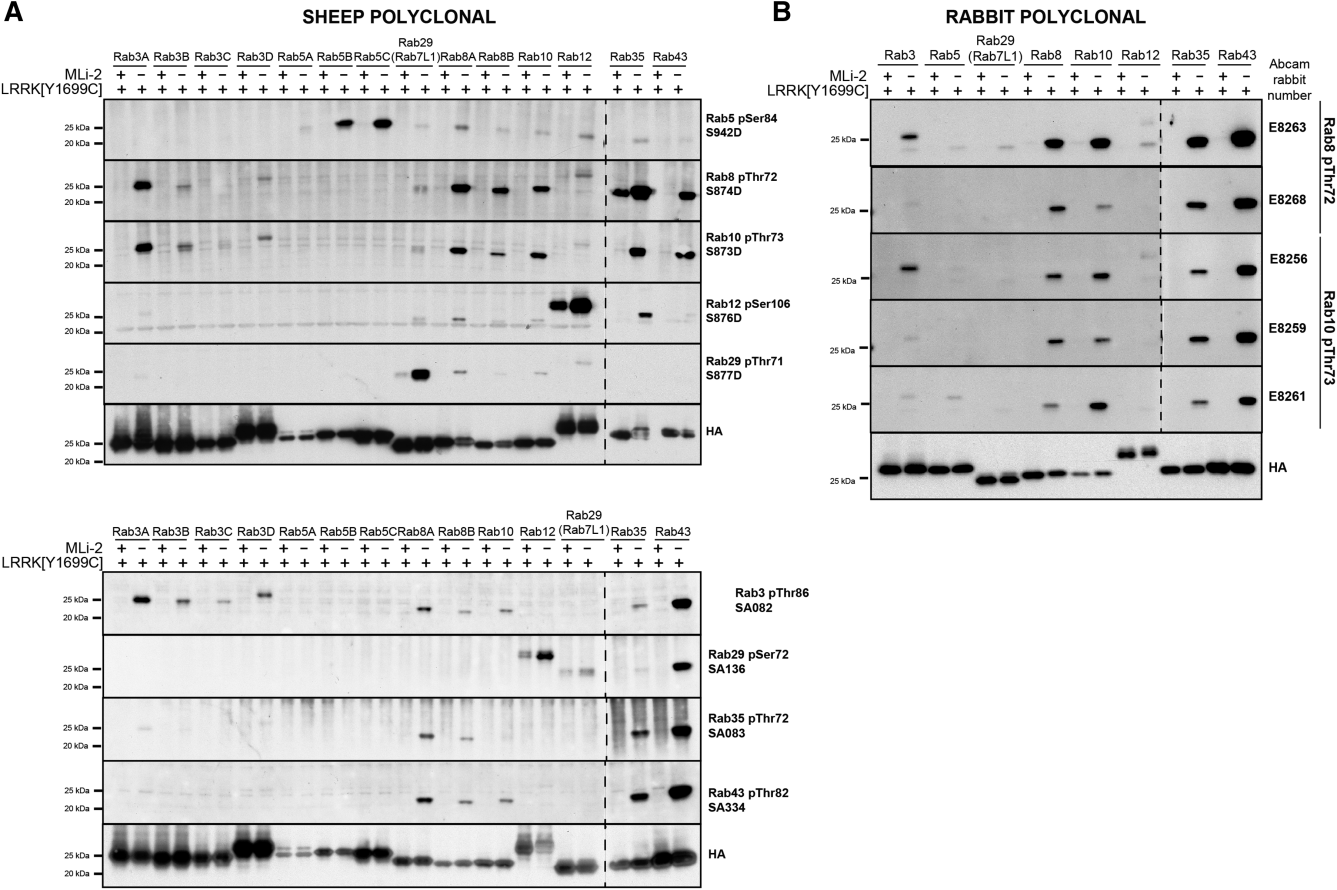
Cross-reactivity of the polyclonal sheep and rabbit phospho-Rab protein antibodies. HEK293 cell extracts overexpressing LRRK2[Y1699C] and the indicated HA-tagged Rab protein were treated ±150 nM MLi-2 for 90 min and then lysed. The cell lysates were immunoblotted with the indicated sheep (**A**) and rabbit (**B**) affinity-purified phospho-specific polyclonal antibodies (all at 1 µg/ml antibody — for only sheep polyclonal antibodies, we also included 10 µg/ml dephospho-peptide antigen). Ten micrograms of whole cell lysate were loaded per lane for the sheep polyclonal antibodies (**A**) and 1 µg of cell lysate per lane for the rabbit polyclonal antibodies (**B**). Immunoblots developed with ECL.

We first raised sheep polyclonal antibodies against phospho-peptides encompassing the LRRK2 phosphorylation site of Rab3, Rab5, Rab8, Rab10, Rab12, Rab29-Thr71, Rab29-Ser72, Rab35 and Rab43 (Figure 2A). For each of the sheep polyclonal antibodies, we immunised animals with a single phospho-peptide encompassing the LRRK2 phosphorylation site, conjugated via an N-terminal Cys residue to a mixture of KLH and bovine serum albumin (Table 1). We also generated rabbit polyclonal antibodies against phospho-peptides encompassing the LRRK2 phosphorylation site on Rab8 and Rab10 (Figure 2B). For the rabbit antibodies, we adopted a novel strategy, in which we immunised each animal with two antigen phospho-peptides (Table 2) encompassing the LRRK2 phosphorylation site that possesses a Cys-aminohexanoic acid moiety either on its N-terminus or C-terminus (Table 2). We also blocked the remaining free terminal residue (acetyl group for N-terminus or amide for C-terminus) (Table 2). These peptides were conjugated to KLH and ovalbumin via the terminal Cys residue prior to immunisation. This strategy was selected to maximise peptide exposure in both possible conjugation orientations and was previously adopted to generate sensitive antibodies recognising PINK1 phosphorylated ubiquitin [28].

Polyclonal antibodies were evaluated after affinity purification on phospho-peptide antigen column, and for rabbit antibodies, additional negative affinity purification on a non-phospho-peptide antigen column was performed. To assess the selectivity of each antibody, we created a panel of different LRRK2-phosphorylated Rab proteins in HEK293 cells, by overexpressing Rab proteins with pathogenic LRRK2[Y1699C]. Prior to lysis, cells were treated with or without 150 nM MLi-2 LRRK2 inhibitor to enable phosphorylation dependence of each antibody to be established (Figure 2). This analysis revealed that all of the polyclonal sheep (Figure 2A) and rabbit (Figure 2B) antibodies recognised the LRRK2-phosphorylated Rab protein to which they were raised against in a MLi-2-sensitive manner. The polyclonal antibodies raised against Rab proteins with a Ser at the LRRK2 phosphorylation site (Rab5 isoforms, Rab12 and Rab29), were more selective than antibodies raised against Thr phosphorylation sites (Figure 2A). For example, Rab5 and Rab12 phospho-specific antibodies only cross-reacted weakly or not at all with other LRRK2-phosphorylated Rab proteins (Figure 2A). The Rab29[Ser72] phospho-antibody cross-reacted with Rab12 and Rab43, but not with other Rab proteins. In contrast, polyclonal antibodies raised against Rab proteins possessing a Thr residue at the phosphorylation site (Rab3, Rab8, Rab10, Rab35 and Rab 43) were not selective and cross-reacted with many other Rab proteins, especially those phosphorylated on Thr residues (Figure 2A,B). The Rab35 and Rab43 phospho-antibodies did not cross-react with LRRK2-phosphorylated Rab3 isoforms, but cross-reacted with all other Thr-phosphorylated Rab proteins. The antibody raised against the Rab29[Thr71] site was also selective (Figure 2A), presumably because it is unique being one residue removed from the other LRRK2 phosphorylation sites. The relative specificity of each of the polyclonal antibodies did not vary significantly between antibody bleeds (Supplementary Figure S1). The rabbit polyclonal antibodies were estimated to be ∼10-fold more sensitive than the sheep antibodies, based on the relative amounts of HEK 293 extract required to detect a strong signal (1 µg for rabbit polyclonal antibody versus 10 µg of HEK293 cell extract for sheep polyclonal antibody) (Figure 2A,B). It is possible that the different peptide immunisation strategies deployed account for the differences in affinity of these antibodies. Further work comparing both strategies in parallel would be required to ascertain this.

### Analysis of endogenous LRRK2-phosphorylated Rab proteins with polyclonal phospho-specific antibodies

**Figure 3.**
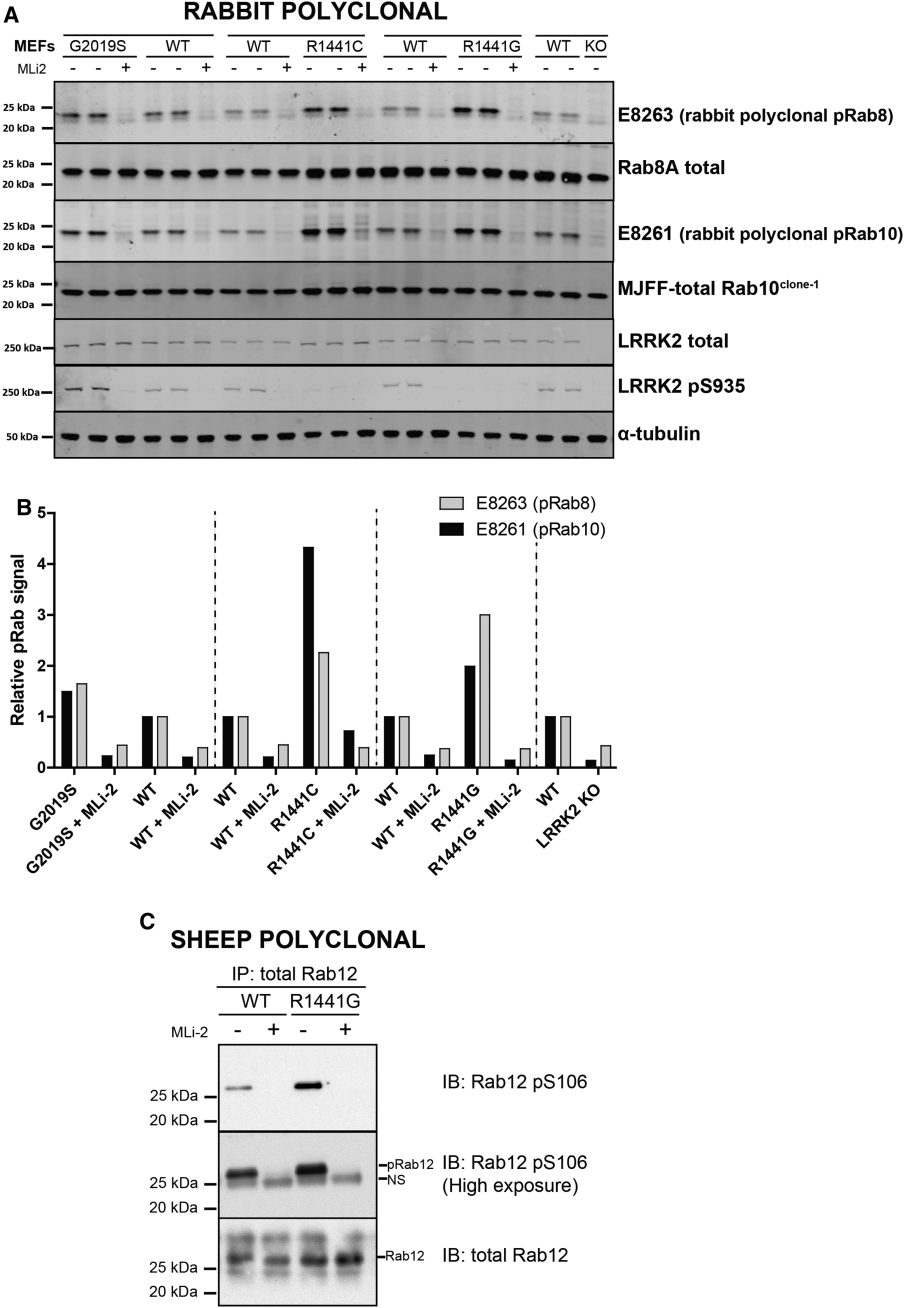
Detection of endogenous LRRK2-mediated Rab protein phosphorylation with polyclonal antibodies. (**A**) The indicated littermate wild-type and knock-in or knock-out MEF cells were treated ±100 nM MLi-2 for 90 min and lysed. The cell lysates (10 µg) were immunoblotted with the indicated antibodies and the membranes developed using the Odyssey CLx Western Blot imaging system (all at 1 µg/ml antibody). Similar results were obtained in two independent experiments. (**B**) Quantitation of immunoblots was undertaken by analysing pRab8/tubulin (**Bi**) or pRab10/tubulin (**Bii**). (**C**) As in (**A**) except that Rab12 was immunoprecipitated using sheep polyclonal total Rab12 antibody from 5 mg of each of the indicated cell extracts. Ninety percent of the immunoprecipitates was immunoblotted with the phospho-Rab12 pSer106 antibody and 1% immunoblotted with the total Rab12 antibody (all at 1 µg/ml antibody and for the phospho-Rab12 pSer106 antibody, 10 µg/ml of dephospho-peptide was also included). Immunoblot (**A**) developed with LI-COR, and immunoblot (**C**) developed with ECL.

The rabbit polyclonal Rab8 and Rab10 phospho-specific antibodies readily detected endogenous Rab protein phosphorylation in MEFs that have previously been used to assess LRRK2-mediated phosphorylation of Rab proteins [10,11] (Figure 3A). Phosphorylation was inhibited by the treatment with MLi-2 or knock-out of LRRK2, confirming that the antibodies are detecting a LRRK2-regulated Rab protein phosphorylation (Figure 3A). Moreover, LRRK2[G2019S], LRRK2[R1441C] and LRRK2[R1441G] knock-in mutations all increased Rab protein phosphorylation 2- to 4-fold, and this increase was detected by either of the rabbit polyclonal Rab8 or Rab10 antibodies (Figure 3A,B). The sheep polyclonal phospho-specific antibodies were insufficiently sensitive to detect endogenous Rab protein phosphorylation by direct immunoblot analysis of whole cell lysates. However, by immunoprecipitating endogenous Rab12 protein and then immunoblotting with phospho-Rab12 sheep polyclonal antibodies, we readily observed phosphorylated Rab12 in MEFs (Figure 3C). Moreover, employing this strategy, a 3-fold increase in the phosphorylation of Rab12 was observed in the LRRK2[R1441G] knock-in MEFs compared with wild-type MEFs that were suppressed by MLi-2 LRRK2 inhibitor treatment (Figure 3C).

### Development and characterisation of exquisitely specific MJFF-pRab10 rabbit monoclonal antibodies

**Figure 4.**
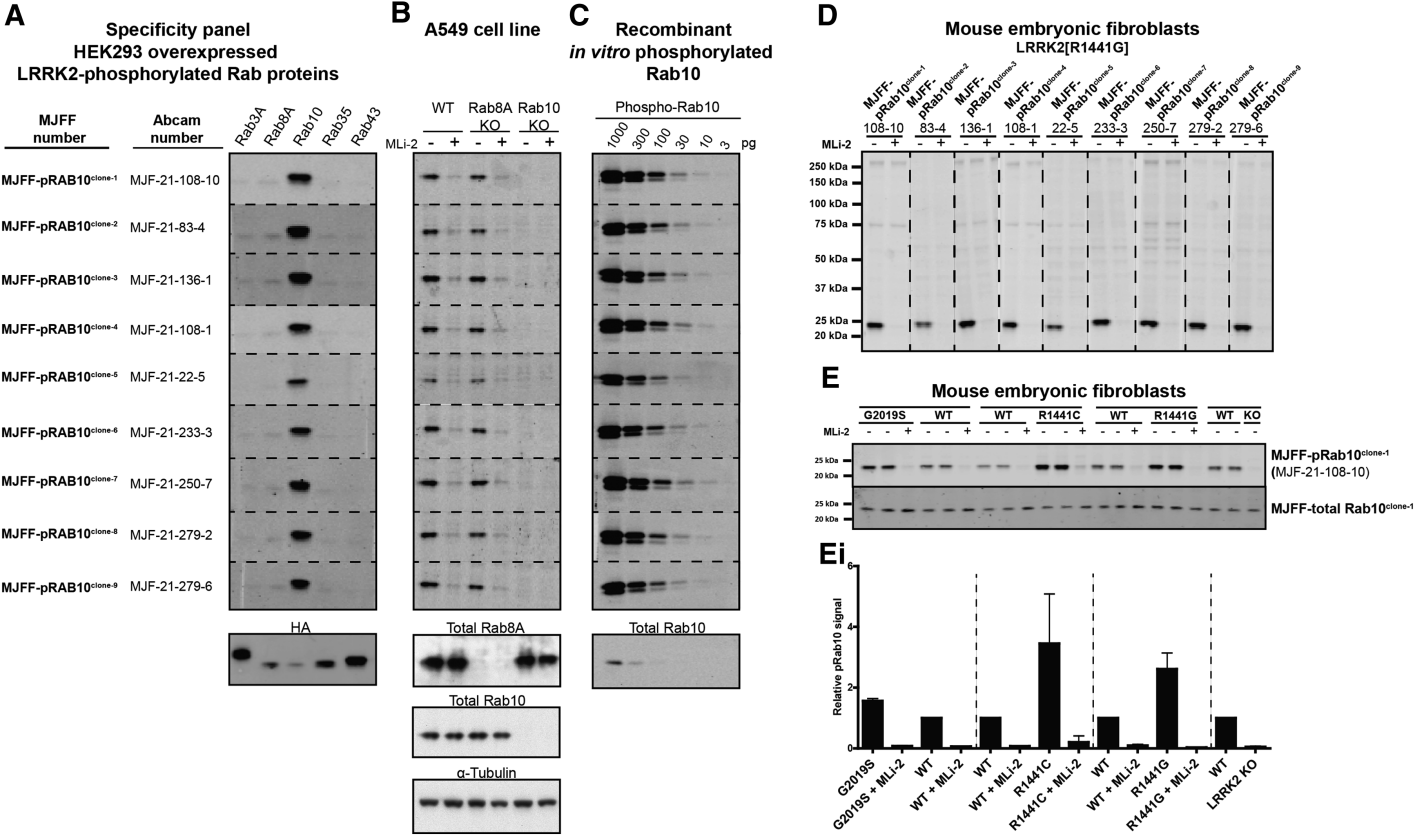
Characterisation of the selectivity and potency of MJFF-pRab10 rabbit monoclonal antibodies. (**A)** HEK293 cell extracts overexpressing LRRK2[Y1699C] and the indicated HA-tagged Rab protein were treated ±150 nM MLi-2 for 90 min and then lysed. The cell lysates (0.1 µg) were immunoblotted with the indicated MJFF-pRab10 hybridoma clones (all at 0.5 µg/ml antibody). (**B**) The indicated wild-type and knock-out A549 cells were treated ±100 nM MLi-2 for 90 min and then lysed. The cell lysates (20 µg) were immunoblotted with the indicated MJFF-pRab10 hybridoma clones as well as shown total antibodies (all at 0.5 µg/ml antibody). (**C**) The indicated amounts of recombinant LRRK2-phosphorylated Rab10 were subjected to immunoblotting with the specified MJFF-pRab10 hybridoma clones as well as the total RAB10 antibody purchased from Cell Signaling Technology (CST) (all at 0.5 µg/ml antibody). (**D**) LRRK2[R1441G] MEFSs were treated ±100 nM MLi-2 for 90 min and then lysed. The cell lysates (5 µg) were immunoblotted with the indicated MJFF-pRab10 hybridoma clones (all at 0.5 µg/ml antibody). (**E**) The indicated littermate wild-type and knock-in or knock-out MEF cells were treated ±100 nM MLi-2 for 90 min and lysed. The cell lysates (5 µg) were immunoblotted with the indicated antibodies and the membranes developed using the Odyssey CLx scan Western Blot imaging system (all at 1 µg/ml antibody). Quantitation of immunoblots was undertaken by analysing pRab10/tubulin (**Ei**). Immunoblots were developed with LI-COR.

In an attempt to raise selective phospho-specific Rab10 protein antibodies, we initiated a programme to generate rabbit monoclonal antibodies. We assessed the selectivity and potency of the top 9 hybridoma clones termed MJFF-pRAB10^clones-1 to -9^. Strikingly, they were all highly selective and did not cross-react with other Thr LRRK2-phosphorylated Rab proteins (Figure 4A). To establish the *in vivo* selectivity of these clones, we utilised previously described wild-type, Rab8A knock-out [12] and Rab10 knock-out [11] A549 cells that express LRRK2, albeit at significantly lower levels than MEFs. All the MJFF-pRAB10 monoclonal antibodies readily detected phosphorylated Rab10 in wild-type and Rab8A knock-out, but not in Rab10 knock-out A549, cells confirming the exquisite specificity of these antibodies (Figure 4B). Treatment with MLi-2 also ablated recognition of phosphorylated Rab10 in A549 cells with all of the MJFF-pRab10 antibodies (Figure 4B). To analyse the potency of each MJFF-pRAB10 monoclonal antibody, we immunoblotted a serial dilution of quantified amounts of LRRK2-phosphorylated Rab10 (Figure 4C). This revealed that the antibodies were exceptionally potent, detecting as little as 10 pg of LRRK2-phosphorylated Rab10 (0.44 fmol). These antibodies were much more potent than the commercial total Rab10 antibody we routinely employ that detects ∼300–1000 pg of Rab10 protein (Figure 4C, lower panel).

The MJFF-pRAB10 also recognised phosphorylated Rab10 in LRRK2[R1441G] knock-in MEFs in a manner that was suppressed by MLi-2 treatment (Figure 4D). The immunoblots were strikingly clean, with the phosphorylated Rab10 being the only major protein in the extract recognised by the MJFF-pRAB10 antibodies (Figure 4D). We immunoblotted a panel of wild-type and LRRK2 mutant MEFs with MJFF-pRAB10^clone-1^, which confirmed that 2- to 3-fold increased phosphorylation of Rab10 was readily observed with pathogenic LRRK2[G2019S], LRRK2[R1441G] and LRRK2[R1441C] knock-in mutations and signal lost with LRRK2 knock-out or MLi-2 treatment (Figure 4E).

**Figure 5.**
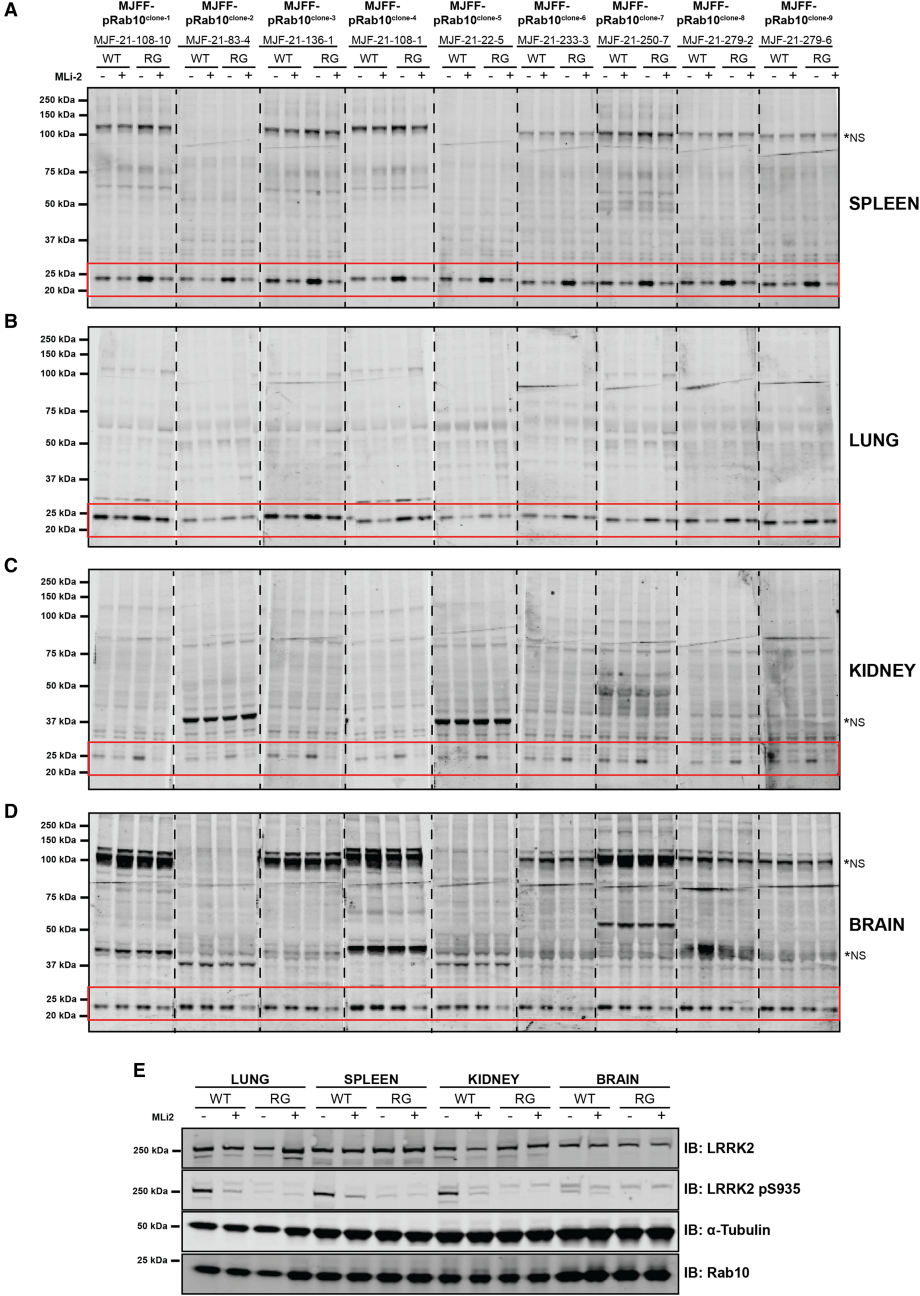
Characterisation of MJFF-pRab10 rabbit monoclonal antibodies in wild-type and LRRK2[R1441G] knock-in mouse tissue extracts. Wild-type and LRRK2[R1441G] knock-in mice were administered with MLi-2 (3 mg/kg) by subcutaneous injection. After 60 min, animals were killed and the spleen (**A**), lung (**B**), kidney (**C**) and brain (**D**) extracts were generated and immunoblotted with the indicated MJFF-pRab10 hybridoma clones (all at 0.5 µg/ml antibody). Thirty micrograms of the extract were immunoblotted for the spleen, lung, kidney and 40 µg for brain. (**E**) The denoted extracts were immunoblotted with the indicated total and phospho-Ser935 LRRK2 antibodies. The regions of the nitrocellulose membrane containing phosphorylated Rab10 are highlighted in a red box, *NS, major non-specific bands. Immunoblots were developed with LI-COR.

We next immunoblotted mouse spleen (Figure 5A), lung (Figure 5B), kidney (Figure 5C) and brain (Figure 5D) extracts derived from littermate wild-type and homozygous LRRK2[R1441G] mice with the MJFF-pRAB10 antibodies. Phosphorylated Rab10 was readily detected in the spleen, lung and kidney extracts and enhanced 2- to 3-fold in LRRK2[R1441G] knock-in tissues compared with wild-type tissues (Figure 5A–C). We also injected mice with an intermediate dose of LRRK2 inhibitor (3 mg/kg MLi-2) that partially reduces LRRK2 activity and Rab10 phosphorylation *in vivo* [11]. Consistent with this, we observed that 3 mg/kg MLi-2 partially suppressed phosphorylation of Rab10 in the lung, spleen and kidney (Figure 5A–C) in addition to the well-studied LRRK2 Ser935 biomarker phosphorylation that is controlled by LRRK2 kinase activity [22,29] (Figure 5E). As observed before, Ser935 is largely ablated by the R1441G knock-in mutation for reasons that are not understood [11,30]. Immunoblotting 40 µg of whole brain extract from wild-type mice revealed only a weak phospho-Rab10 band that was not reduced by MLi-2 treatment (Figure 5D). Only a weak Ser935 phosphorylation signal was observed in the wild-type brain which was suppressed by MLi-2 (Figure 5E). In the brain extract derived from LRRK2[R1441G] knock-in animals, the signal of phosphorylated Rab10 was similar to that observed in wild-type mice, but was partially reduced by MLi-2 treatment (Figure 5D).

With tissues, the immunoblots were not as clean as observed with fibroblasts (Figure 4D), but phosphorylated Rab10 was still a major band especially in the spleen (Figure 5A) and lung (Figure 5B). MJFF-pRAB10^clones-1 and -2^ were selected for recombinant antibody production as these were judged to be the most potent and selective antibodies.

**Figure 6.**
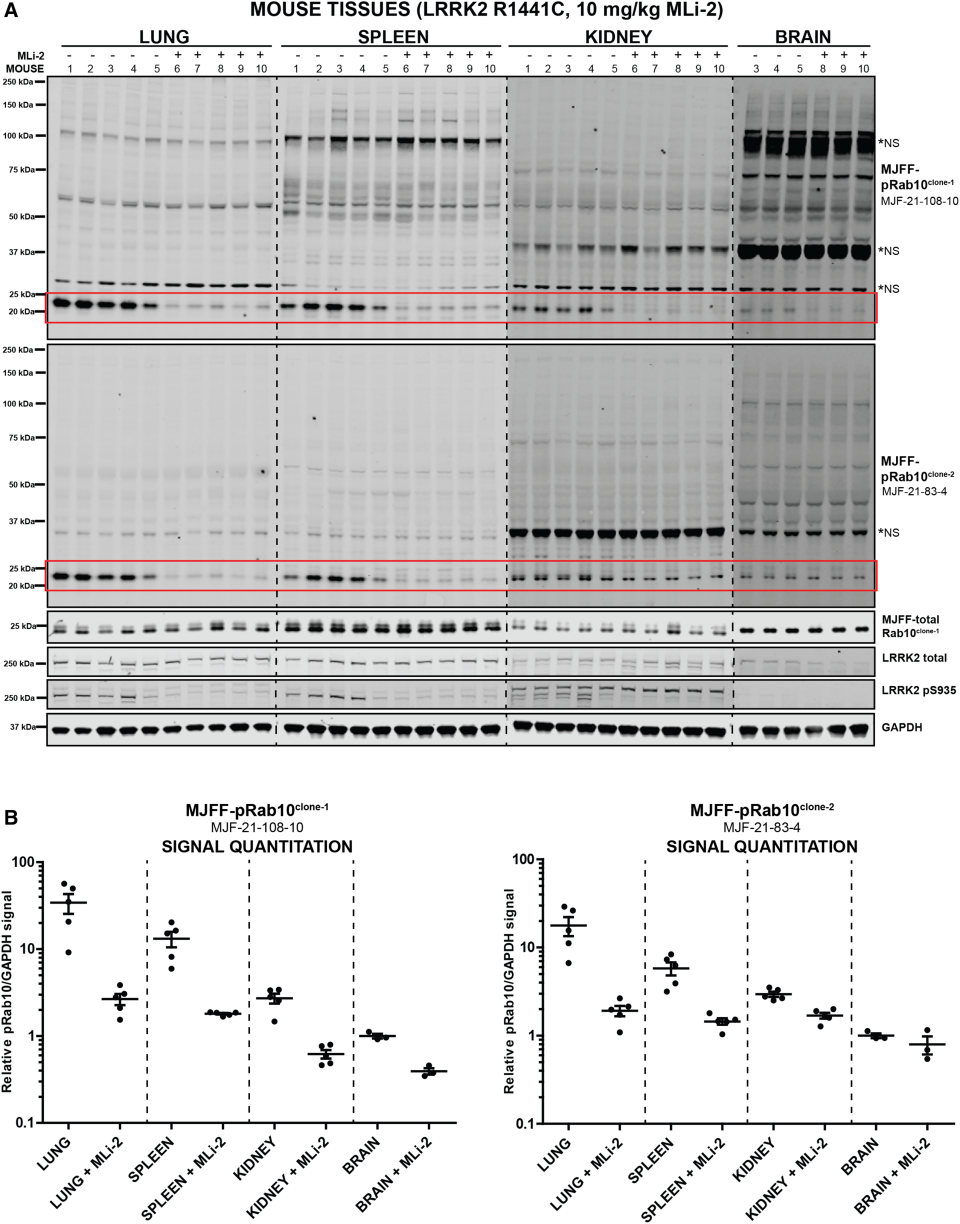
Characterisation of MJFF-pRab10^clone-1^ and MJFF-pRab10^clone-2^ rabbit monoclonal antibodies in LRRK2[R1441C] knock-in mouse tissue extracts. LRRK2[R1441C] knock-in mice were administered with MLi-2 (10 mg/kg) by subcutaneous injection. After 60 min, animals were killed, and the lung, spleen, kidney and brain extracts were generated and immunoblotted with the indicated MJFF-pRab10 hybridoma clones (at 1 µg/ml antibody). Forty micrograms of the extract were immunoblotted for the spleen, lung, kidney and brain (**A**). Quantitation of immunoblots was undertaken by analysing pRab10/GAPDH (**B**). The regions of the nitrocellulose membrane containing phosphorylated Rab10 are highlighted in a red box. *NS, major non-specific bands. Immunoblots were developed with LI-COR.

To ensure that the recombinant pRAB10^clone-1^ and pRAB10^clone-2^ antibodies worked as well as original hybridoma antibodies, we immunoblotted the lung, spleen, kidney and brain tissues of LRRK2[R1441C] knock-in mice treated ±10 mg/kg MLi-2 with recombinant antibody (Figure 6). This confirmed that phosphorylated Rab10 was readily detected in order of strength in the lung (best), then the spleen, kidney and brain (weakest) (Figure 6A,B). MJFF-pRAB10^clone-1^ is more potent than MJFF-pRAB10^clone-2^ and readily detects phosphorylated Rab10 in all four tissues (Figure 6A,B). MJFF-pRAB10^clone-2^ has a lower background on immunoblot analysis and clearly detects phosphorylated Rab10 in the lung and spleen with significantly less background than the MJFF-pRAB10^clone-1^ antibody (Figure 6A). However, MJFF-pRAB10^clone-2^ antibody is less sensitive than MJFF-pRAB10^clone-1^ antibody at recognising phosphorylated Rab10 in the kidney and brain (Figure 6A,B). Both antibodies worked equally well in detecting LRRK2-phosphorylated Rab10 in MEFs and A549 cells (Figure 4). Owing to its lower background, MJFF-pRAB10^clone-2^ may be better than MJFF-pRAB10^clone-1^ in many cell lines and in the lung and spleen. However, if the signal of phosphorylated Rab10 is weak, as it is for the kidney and brain in wild-type mice, we would recommend using pRAB10^clone-1^ antibody.

### Analysis of Rab10 phosphorylation in the human brain cingulate cortex samples

**Figure 7.**
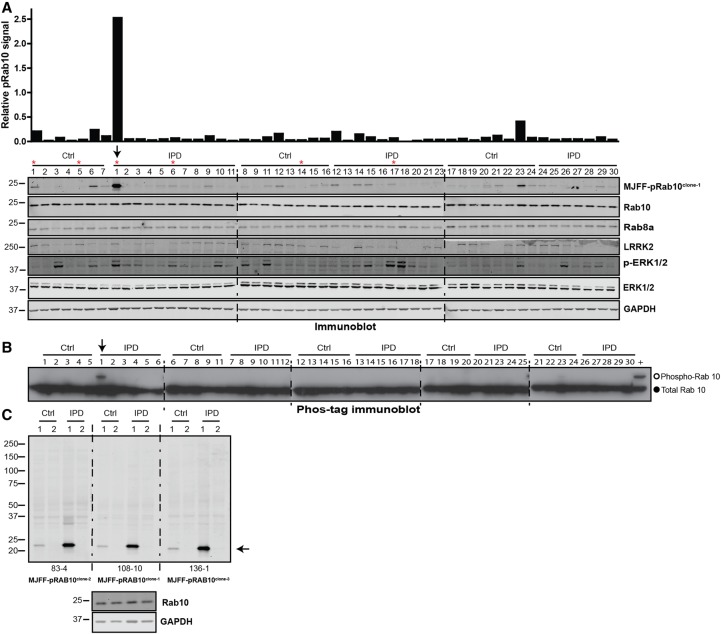
Phosphorylation of Rab10 in control and idiopathic Parkinson's disease patients' cingulate cortex. (**A**) Cingulate cortex samples derived from Control (Ctrl) and idiopathic Parkinson's disease (IPD) donors were obtained from the Queen Square Brain Bank (UCL). Extracts from each of these were generated and 20 µg subjected to immunoblot analysis using the indicated antibodies. Donors marked with an asterisk are over 90 years of age. The sample marked with an arrow (IPD donor 1) displays the highest levels of Rab10 phosphorylation. (**B**) As in (**A**) except that samples were subjected to Phos-tag polyacrylamide gel electrophoresis prior to immunoblot analysis employing a total Rab10 antibody. Cell lysates prepared from LRRK2[R1441C] knock-in MEFs were used as a positive control (+) for Rab10 phosphorylation and run in the last lane. The open circles on the Phos-tag gel correspond to the position that the phosphorylated Rab protein migrates and the closed circle the position that the dephosphorylated Rab protein migrates. (**C**) Control (donors 1 and 2) and IPD samples (donors 1 and 2) were subjected to immunoblot analysis using the MJFF-pRab10^clone-1–3^ monoclonal antibodies. The arrow indicates the band corresponding to the phospho-Rab10 signal. Immunoblots were developed with LI-COR.

To determine whether MJFF-pRAB10 monoclonal antibodies detect phosphorylated Rab10 in human brain, we obtained from the Queen Square Brain Bank (UCL), cingulate cortex samples from 23 controls and 28 idiopathic Parkinson's patients. Protein lysate (2–10 mg) was obtained from each tissue sample. Twenty micrograms of brain extracts derived from each sample was immunoblotted with the monoclonal MJFF-pRAB10^clone-1^ antibody, revealing that levels of phosphorylated Rab10 were very low or non-detectable in the majority of samples (Figure 7A). However, phosphorylated Rab10 was elevated in a few samples, especially in one of the idiopathic Parkinson's samples (donor 1, derived from a 91-year-old female patient with no known mutations) (Figure 7A). The panel contained five other cingulate cortex samples from patients over 90 years of age which did not display elevated levels of phosphorylated Rab10 (Figure 7A). pERK1/2 antibody was used to analyse the general stability of phosphorylation across the samples. We observed the variability of ERK1/2 phosphorylation levels with high levels seen in five controls and six to seven idiopathic samples (Figure 7A). There was no correlation between Rab10 phosphorylation and ERK1/2 phosphorylation. In contrast, the levels of total ERK1/ERK2 were very similar in most samples (Figure 7A). We also attempted to analyse LRRK2 Ser935 phosphorylation and Akt Ser473 phosphorylation, but this was found to be undetectable for all samples. We also analysed the cingulate cortex samples employing phos-tag immunoblotting analysis, in which retardation of the phosphorylated Rab10 on a phos-tag polyacrylamide gel was monitored with a total Rab10 antibody (Figure 7B). Using this approach, only the same sample from the 91-year-old female Parkinson's patient displayed significant levels of phosphorylated Rab10 (Figure 7B).

The total levels of Rab10 and Rab8A were fairly constant between the cingulate cortex samples (Figure 7A). LRRK2 levels were much lower and varied significantly, and were not detectable in a few samples (Figure 7A). It is possible that LRRK2 being a large protein is more susceptible to post-mortem/long-term storage degradation compared with Rab proteins. The sample from idiopathic Parkinson's donor 1 that displayed highest levels of phosphorylated Rab10 had an intermediate level of LRRK2 (Figure 7A). We also subjected two controls and two idiopathic cingulate cortex samples (one with higher Rab10 phosphorylation and one with lower Rab10 phosphorylation) to immunoblot analysis with the MJFF-pRAB10^clones-1 to -3^ monoclonal antibodies (Figure 7C). The immunoblots with all antibodies were remarkably clean, displaying only one major protein corresponding to phosphorylated Rab10 (Figure 7C).

### Development and characterisation of pan-selective MJFF-pRab8 rabbit monoclonal antibodies

**Figure 8.**
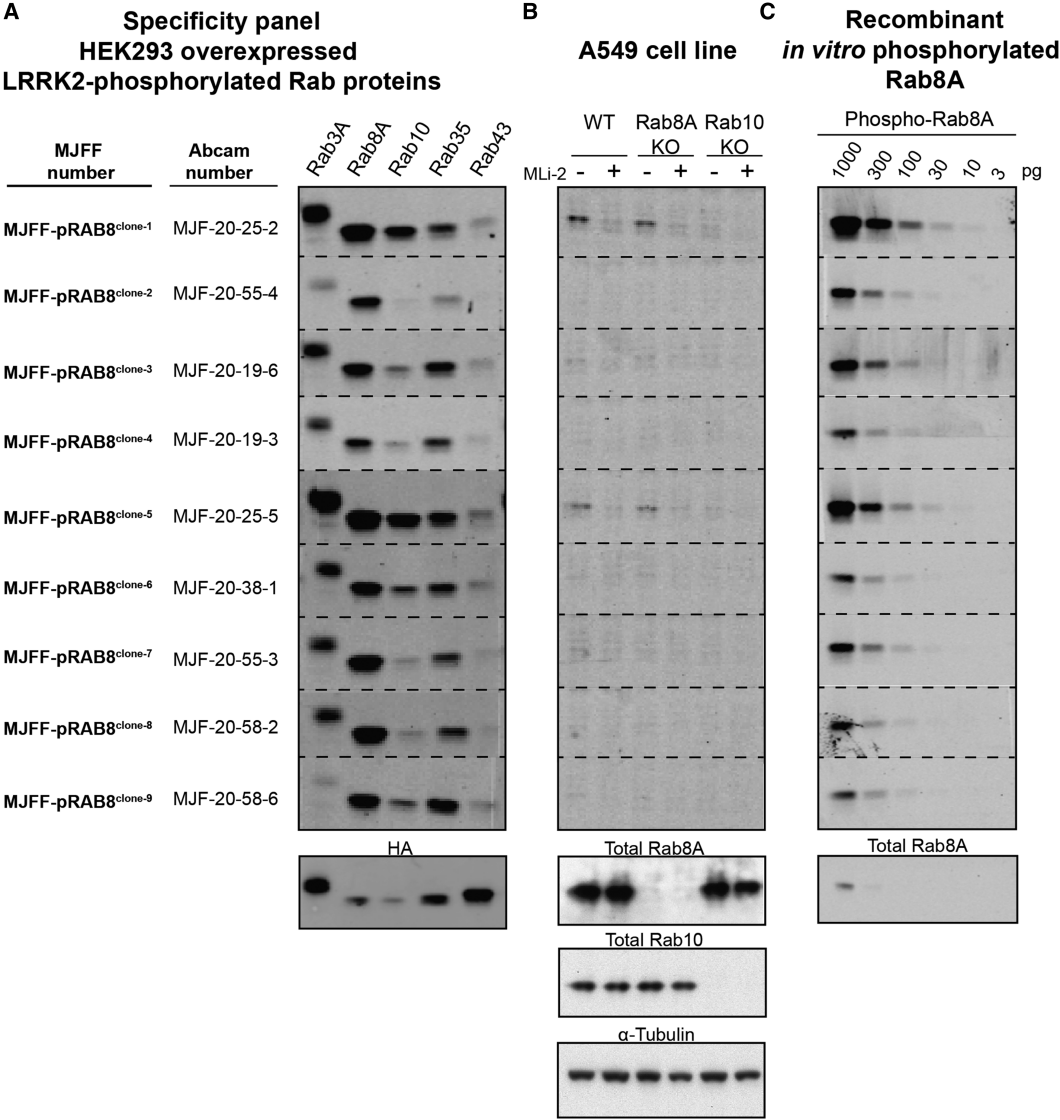
Characterisation of the selectivity and potency of MJFF-pRab8 rabbit monoclonal antibodies. (**A**) HEK293 cell extracts overexpressing LRRK2[Y1699C] and the indicated HA-tagged Rab protein were treated ±150 nM MLi-2 for 90 min and then lysed. The cell lysates (0.1 µg) were immunoblotted with the indicated MJFF-pRab8 hybridoma clones (all at 0.5 µg/ml antibody). (**B**) The indicated wild-type and knock-out A549 cells were treated ±100 nM MLi-2 for 90 min and then lysed. The cell lysates (20 µg) were immunoblotted with the specified MJFF-pRab8 hybridoma clones and the denoted total antibodies (all at 0.5 µg/ml antibody). (**C**) The shown amounts of recombinant LRRK2-phosphorylated Rab8A were subjected to immunoblotting with the indicated MJFF-pRab8 hybridoma clones as well as the total RAB8A antibody purchased from Cell Signaling Technology (CST) (all at 0.5 µg/ml antibody). Immunoblots were developed with LI-COR.

We also raised rabbit monoclonal phospho-antibodies for LRRK2-phosphorylated Rab8 and assessed the selectivity and potency of the top 9 hybridoma clones termed MJFF-pRAB8^clones-1 to -9^ (Figure 8). In contrast to Rab10, none of the MJFF-pRAB8 monoclonal antibodies were selective for LRRK2-phosphorylated Rab8A and clearly recognised other LRRK2-phosphorylated Rab proteins with similar potency to Rab8A (Figure 8A). MJFF-pRAB8^clone-2^ was the most selective, but still recognised other LRRK2-phosphorylated Rab proteins. Two MJFF-pRAB8 antibodies detected a phosphorylated Rab species in wild-type A549 cells that were ablated with LRRK2 inhibitors (MJFF-pRAB8^clones-1 and -5^). However, these antibodies are cross-reacting with LRRK2-phosphorylated Rab10, as signal was lost with the Rab10 knock-out, but not with the Rab8A knock-out (Figure 8B). We also immunoblotted a serial dilution of LRRK2-phosphorylated Rab8A with the selected MJFF-pRAB8 antibodies, revealing that most of the Rab8 monoclonal antibody clones detected ∼30 pg of LRRK2-phosphorylated Rab8 (1.27 fmol) (Figure 8C). The MJFF-pRAB8 antibodies are therefore moderately less potent than the MJFF-pRAB10 antibodies in immunoblot analysis (compare Figures 4C and 8C), but still much more potent than the commercial total Rab8 antibody that detects only ∼1000 pg of Rab8A (Figure 8C, lower panel). MJFF-pRAB8^clones-1 to -2^ were selected for recombinant protein production as Clone-1 was the most potent and Clone-2 the most selective.

**Figure 9.**
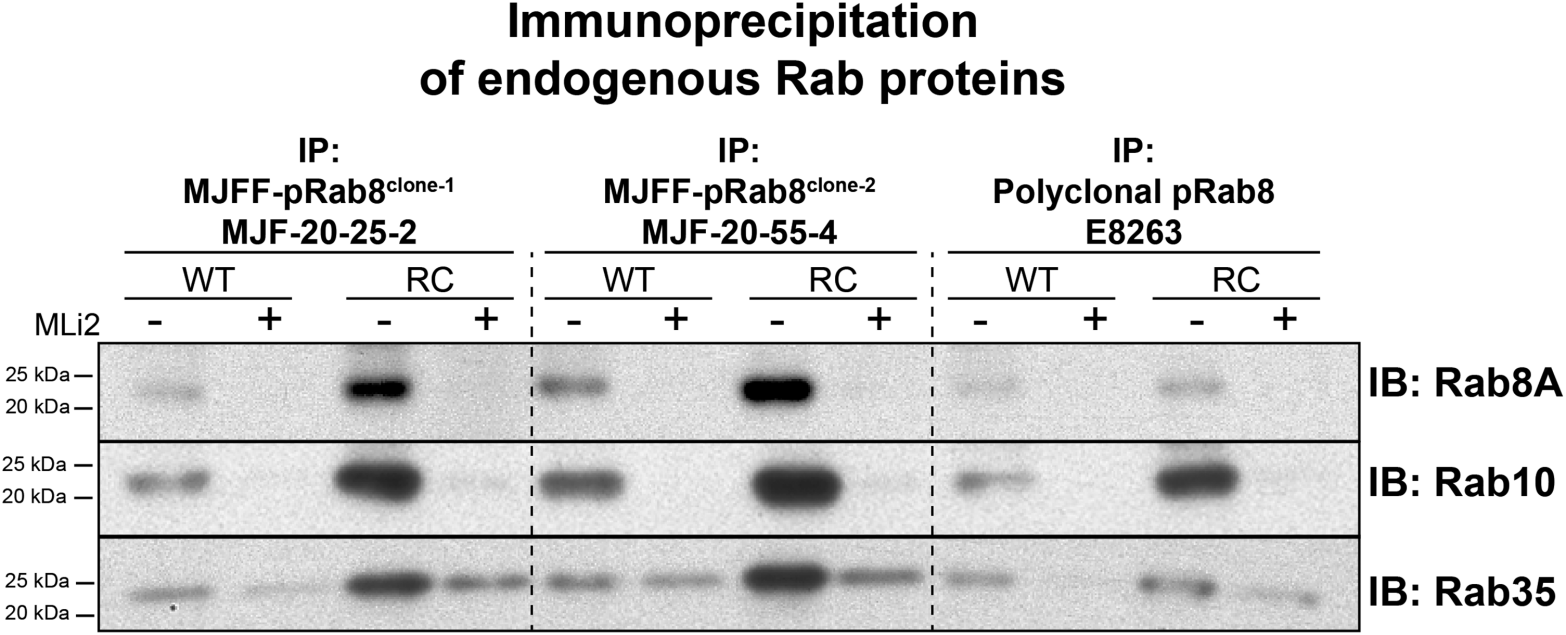
The pan-selective MJFF-pRab8 antibodies immunoprecipiate various LRRK2-phosphorylated Rab proteins. Littermate wild-type and LRRK2[R1441C] knock-in MEFs were treated ±100 nM MLi-2 for 90 min and lysed. The cell lysates (5 mg) were subjected to immunoprecipitation using the indicated MJFF-pRab8 monoclonal antibodies (20 µg) or rabbit polyclonal antibody (E8263) (50 µg). Forty percent of the immunoprecipitates was immunoblotted with total Rab43 antibody, 40% with total Rab35 antibody, 10% with Rab8A antibody and 10% with total Rab10 antibody (all at 1 µg/ml antibody). The specificities of the total Rab8A, Rab10 and Rab35 have been previously validated using appropriate A549 CRISPR/CAS9 knock-out cell extracts [12]. Immunoblots were developed with ECL.

We next explored whether it was possible to exploit the lack of selectivity of the MJFF-pRAB8 monoclonal antibodies to immunoprecipitate diverse sets of LRRK2-phosphorylated endogenous Rab proteins. Immunoprecipitations were undertaken using the MJFF-pRAB8^clones-1 and -2^ monoclonal antibodies, in parallel with the polyclonal antibodies, on cell extracts derived from wild-type and LRRK2[R1441C] knock-in MEFs treated with or without 100 nM MLi-2 prior to cell lysis. The immunoprecipitates were then subjected to immunoblot analysis with various total Rab protein antibodies. This revealed that MJFF-pRAB8^clones-1 and -2^ and the polyclonal antibodies immunoprecipitated Rab8A in addition to Rab10 and Rab35 (Figure 9). Moreover, levels of all of these proteins were higher in LRRK2[R1441C] MEFs compared with wild-type MEFs, consistent with enhanced phosphorylation of these substrates in the knock-in cells (Figure 9). Furthermore, treatment with MLi-2 prevented the immunoprecipitation of investigated Rab proteins with the MJFF-pRAB8 antibodies, confirming that their immunoprecipitation with the phospho-Rab8 antibody is dependent on LRRK2 phosphorylation (Figure 9). Significantly increased levels of Rab8A, Rab10 and Rab35 were immunoprecipitated with the MJFF-pRAB8^clones-1 and -2^ compared with the polyclonal antibodies (Figure 9), suggesting that the monoclonal antibodies will be more powerful reagents to immunoprecipitate LRRK2-phosphorylated Rab proteins than the polyclonal antibodies.

### Comparison of conventional immunoblotting and Phos-tag protocols to study LRRK2-mediated phosphorylation of Rab proteins

**Figure 10.**
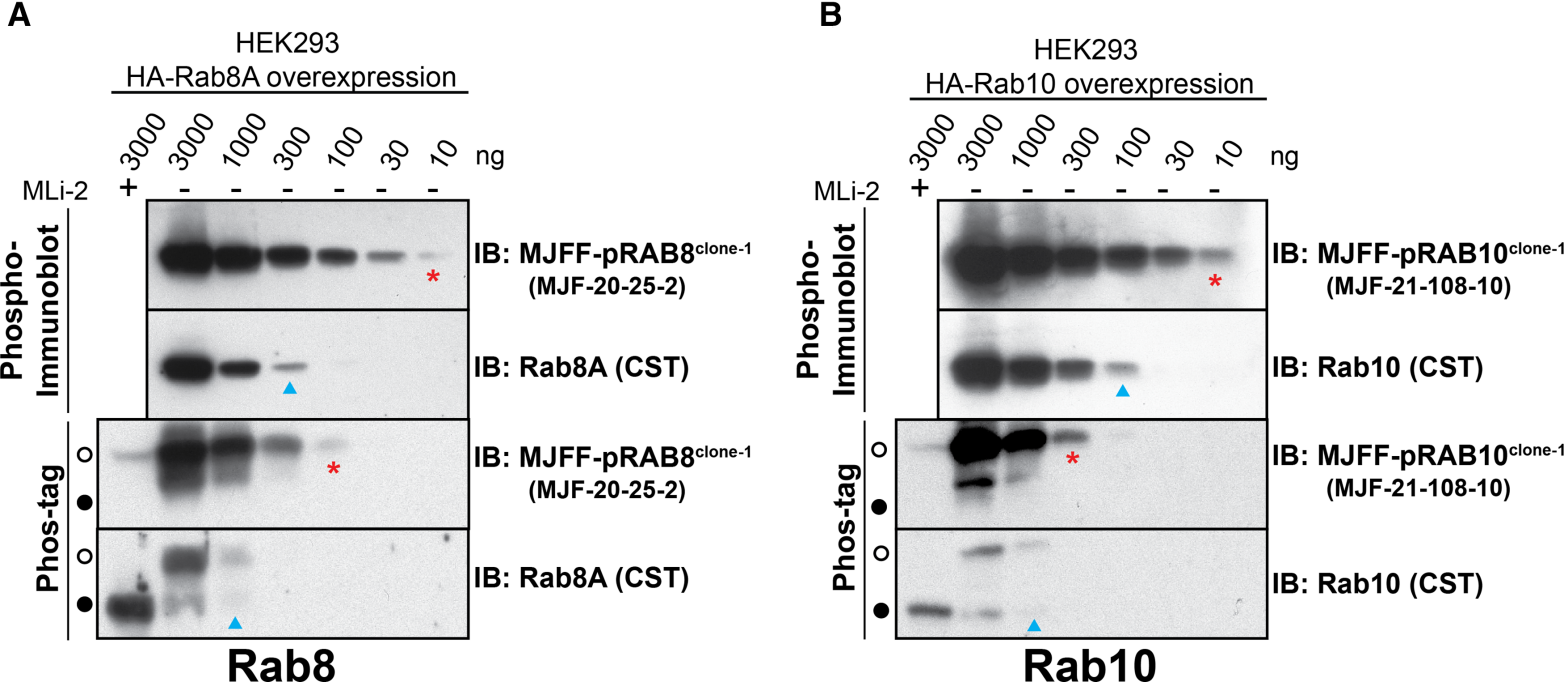
Comparison of the phospho-immunoblotting and Phos-tag protocols to study LRRK2-mediated phosphorylation of Rab proteins. (**A**) HEK293 cell extracts overexpressing LRRK2[Y1699C] and the indicated HA-Rab8A (**A**) or HA-Rab10 (**B**) were treated ±150 nM MLi-2 for 90 min and then lysed. The indicated amounts of cell lysates were subjected to either conventional polyacrylamide gel electrophoresis (upper panel) or Phos-tag polyacrylamide gel electrophoresis (lower panel). Proteins on both gels were transferred to nitrocellulose and subjected to identical immunoblot analysis with indicated antibodies (all at 0.5 µg/ml antibody). The open circles on the Phos-tag gel correspond to the position that phosphorylated Rab protein migrates, and the closed circle to the position that the dephosphorylated Rab protein migrates. Immunoblots were developed with ECL.

Finally, we compared the relative potency of the MJFF-pRAB8 and MJFF-pRAB10 rabbit monoclonal antibodies in a phospho-immunoblot assay with the Phos-tag assay [11]. This was achieved by overexpressing Rab8A (Figure 10A) or Rab10 (Figure 10B) with LRRK2[Y1699C] in HEK293 cells to induce >50% phosphorylation of these Rab proteins. A serial dilution of these extracts was subjected to electrophoresis on either a normal polyacrylamide gel (upper panel) or a Phos-tag polyacrylamide gel (lower panel). Both gels were subjected to immunoblot analysis with the same phospho- and total Rab protein antibodies to enable direct comparison of electrophoresis methods. This revealed that for Rab8A, the conventional immunoblotting assay was ∼3-fold (total antibody) to 10-fold (phospho-antibody) more sensitive than the Phos-tag method (Figure 10A). For Rab10, the conventional immunoblotting assay was ∼10-fold (total antibody) to 30-fold (phospho-antibody) more sensitive than the Phos-tag method (Figure 10A).

## Discussion

As a previous work indicated that Rab10 is a key substrate for LRRK2 [10–13], we prioritised generating a selective antibody recognising endogenous LRRK2-phosphorylated Rab10. This proved more challenging than anticipated, as polyclonal antibodies were not selective. However, by generating rabbit monoclonal antibodies and undertaking selectivity screening at each stage of the hybridoma clone selection process, we succeeded in elaborating antibodies that displayed exquisite selectivity for LRRK2-phosphorylated Rab10 and that were also very potent. Inspection of the amino acid sequences surrounding the Thr73 LRRK2 phosphorylation site on Rab10 suggests that the preceding His72 is unique and not found on other Rab proteins containing a Thr residue phosphorylation site (several Ser-phosphorylated Rab proteins have a preceding His residue) (Figure 1). We speculate that the unique His-Thr(P) motif in Rab10 accounts for the MJFF-pRab10 antibody selectivity.

The MJFF-pRAB10 monoclonal antibodies readily detect endogenous LRRK2-phosphorylated Rab10 in immunoblot analysis of 5 µg of MEF extract (express high levels of LRRK2), 20 µg of A549 cell lysate (express low levels of LRRK2), 30 µg of mouse tissue extract (the lung, spleen and kidney) and 20 µg of the human brain cingulate cortex (Figures 4–7). The LRRK2-phosphorylated Rab10 is the major band in most of these immunoblot analyses that are also relatively clean with remarkably little background (Figures 4–7). Using the MJFF-pRab10 antibodies, we can readily quantify 2–4-fold increases in phosphorylation of Rab10 in MEFs or tissues derived from LRRK2[G2019S] and LRRK2[R1441G/C] knock-in mice (Figures 4–6). We demonstrate in an accompanying manuscript that the phospho-specific MJFF-pRAB10 antibodies can also readily detect LRRK2-mediated phosphorylation of Rab10 in human-derived neutrophils [23].

The experiments using human brain samples (Figure 7) were designed to evaluate whether the MJFF-pRAB10 antibodies could be employed to detect Rab10 phosphorylation in human brain for the first time. We used cingulate cortex samples, as this is more readily available than substantia nigra. It has the advantage of providing several milligrams of proteins, which was required to optimise the assay for the detection of phospho-Rab10 in human brain (Figure 7). Furthermore, even though Parkinson's disease is mainly characterised by degeneration of dopaminergic neurones in substantia nigra, other regions of the brain, including the cingulate cortex, are affected by the disease and are therefore interesting to study [31]. In future work, it would be very interesting to use the MJFF-pRAB10 phospho-specific antibody to investigate Rab10 phosphorylation levels in brain samples derived from LRRK2-mutated Parkinson's disease patients. It will be particularly interesting to perform these experiments in all brain regions available, including substantia nigra and striatum, to determine how Rab10 phosphorylation levels correlate with the genetic mutations and pathology.

A previous mass spectrometry analysis indicated that Rab8 is also one of the major LRRK2-phosphorylated Rab proteins in MEFs and mouse brain extract [10,12]. It would therefore be important to develop a selective antibody that detects LRRK2-phosphorylated Rab8. However, our work indicates that it will be challenging to obtain a selective phospho-Rab8 antibody, likely due to the high sequence similarity surrounding the LRRK2 phosphorylation site on Rab8 and other Rab proteins (Figure 1). Nevertheless, we believe that the pan-selective MJFF-pRAB8 antibodies will still have considerable utility due to their ability to immunoprecipitate a subset of LRRK2-phosphorylated Rab proteins which can then be detected with highly specific total Rab protein antibodies. Thus far, we have shown that this approach works for detecting LRRK2-phosphorylated Rab8A, Rab10 and Rab35, but it is likely based on immunoblotting data (Figure 8A) that other Rab proteins including Rab3 and Rab43 could be also analysed using this approach. We have recently exploited the less-sensitive phospho-Rab8 rabbit polyclonal antibodies to immunoprecipitate and analyse LRRK2-phosphorylated Rab proteins by mass spectrometry that revealed Rab3A and Rab43, in addition to many other Rab proteins that are not phosphorylated by LRRK2 [12].

In future work, it would be interesting to crystallise the MJFF-pRAB8 antibodies with LRRK2-phosphorylated Rab8A protein or a phospho-peptide antigen encompassing the Thr72 phosphorylation site to determine whether it would be possible to engineer enhanced specificity into the MJFF-pRAB8 antibodies. Another possibility would be to immunise rabbits with LRRK2-phosphorylated Rab8A protein rather than phospho-peptide antigen, which may help with generating selective phospho-specific Rab8 antibodies or antibodies that detect a LRRK2 phosphorylation-induced conformation of Rab8A.

Our observations suggest that it may be easier to generate selective antibodies that detect Ser-phosphorylated Rab proteins such as Rab5 and Rab12, as the polyclonal antibodies raised against these LRRK2-phosphorylated Rab proteins are more selective than those recognising Thr-phosphorylated Rab proteins (Figure 2). In a previous work using mass spectrometry, LRRK2-phosphorylated Rab12 was detected in MEFs and mouse brain [10]. We also detected LRRK2-phosphorylated Rab12 after immunoprecipitation of endogenous Rab12 in MEFs using sheep polyclonal phospho-specific Rab12 antibodies (Figure 3C). LRRK2-phosphorylated Rab12 was also observed in human peripheral blood mononuclear cells [13]. We are in the process of generating a rabbit monoclonal phospho-Rab12 Ser106 antibody that, we anticipate, will complement the selective Rab10 phospho-specific antibodies to assess LRRK2 *in vivo* activity. Furthermore, the phospho-Rab12 selective antibodies may be particularly useful for detecting LRRK2 activity in the brain where LRRK2-phosphorylated Rab10 was not easily detected by either immunoblot analysis (Figure 6D) or by mass spectrometry in a previous work [10]. In future work, it will be important to investigate in more depth which Rab proteins are the major LRRK2 substrates in neuronal tissue, a task that will be greatly aided by having access to a wide range of phospho-specific antibodies that detect phosphorylated Rab proteins.

Our data also demonstrate that the conventional phospho-immunoblot assay is more sensitive than the Phos-tag assay in detecting LRRK2-phosphorylated Rab8A and Rab10 (Figure 10). This is partially due to the lower sensitivity of the total Rab8 and Rab10 antibodies that only detects ∼1000 pg of recombinant protein compared with 30–10 pg for the phospho-Rab10 antibody (Figures 4C and 8C). Our data are also consistent with some general loss of both non-phosphorylated and phosphorylated Rab protein associated with the Phos-tag method (Figure 10). The reasons for this are not clear, but it is possible that the electrophoresis and subsequent transfer of Rab proteins from a phos-tag gel to nitrocellulose membrane are less efficient than the conventional polyacrylamide gel and immunoblotting method. Consistent with reduced sensitivity of the Phos-tag assay, we also found it challenging to detect LRRK2 phosphorylation of Rab10 in human neutrophils deploying the Phos-tag method [23]. We would therefore recommend that for future work, conventional phospho-immunoblot assay is deployed as the primary method to evaluate LRRK2-mediated Rab protein phosphorylation. However, in order to assess the stoichiometry of Rab protein phosphorylation, for example to demonstrate high stoichiometry of Rab10 phosphorylation in a patient sample, as we have done for human brain cingulate cortex samples (Figure 7B), the Phos-tag assay still has considerable utility.

In summary, we provide a framework of how to develop and characterise phospho-specific antibodies that recognise LRRK2-phosphorylated Rab proteins. We describe selective phospho-Rab10 (MJFF-pRAB10) and pan-selective phospho-Rab8 (MJFF-pRAB8) antibodies that will greatly help the research community in analysing endogenous LRRK2-mediated phosphorylation of Rab proteins. In addition, our initial work suggests that it should be possible to develop selective antibodies recognising LRRK2-phosphorylated Rab5, Rab12 and Rab29. We also provide the first evidence that the phospho-specific Rab10 antibody can be employed to detect elevated phosphorylation of Rab10 in the human brain sample derived from Parkinson's disease patients. In future work, it will be important to evaluate whether these antibodies work in additional applications such as immunohistochemistry, ELISA assay and fluorescence-activated cell sorting. The phospho-specific antibodies described in the present study will not only help researchers better analyse LRRK2 biology, but also have the potential to be deployed as future biomarkers to monitor LRRK2 activity and the impact of inhibitors in future clinical trials.

## Abbreviations

BSA: bovine serum albumin
ELISA: enzyme-linked immunosorbent assay
ERK1: extracellular signal-regulated kinase-1
HA: human influenza hemagglutinin; HRP, horseradish peroxidase
KLH: keyhole limpet haemocyanin
LRRK2: leucine-rich repeat protein kinase 2
MEFs: mouse embryonic fibroblasts
MLi-2: Merck LRRK2 inhibitor
ROC/COR: Ras of complex proteins C-terminal of Roc domain
TAK1: transforming growth factor beta-activated kinase 1
