# Supplementary material for: Development of phospho-specific Rab protein antibodies to monitor in vivo activity of the LRRK2 Parkinson's disease kinase
Source: Biochem J. 2018 Jan 2;475(1):1–22. doi: 10.1042/BCJ20170802 (PMC5748839; doi:10.1042/BCJ20170802)
Supplement: Supplementary Figure S1 [file BCJ-475-1-s1.pdf]

Supplementary Figure 1

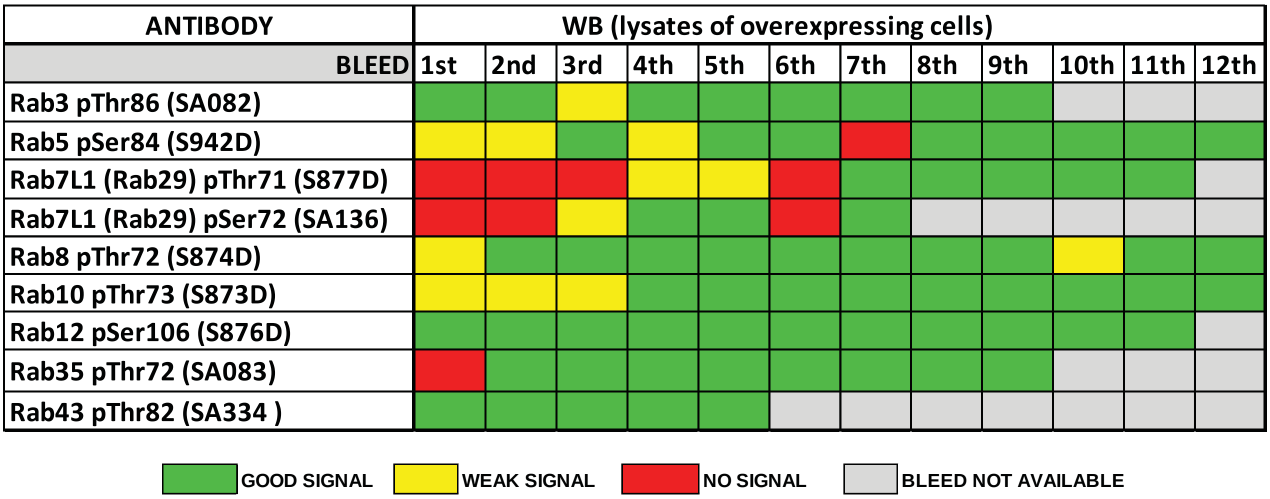

Supplementary Figure 1. Summary of performance of sheep polyclonal antibodies described in Table 1. All bleeds of each antibody were evaluated by immunoblot analysis and ranked by the indicated scoring system.
